# Genome-wide analysis of the peanut *CaM/CML* gene family reveals that the *AhCML69* gene is associated with resistance to *Ralstonia solanacearum*

**DOI:** 10.1186/s12864-024-10108-5

**Published:** 2024-02-21

**Authors:** Dong Yang, Ting Chen, Yushuang Wu, Huiquan Tang, Junyi Yu, Xiaoqiu Dai, Yixiong Zheng, Xiaorong Wan, Yong Yang, Xiaodan Tan

**Affiliations:** Guangzhou Key Laboratory for Research and Development of Crop Germplasm Resources, Zhongkai University of Agriculture and Engineering, Guangzhou, 510225 Guangdong China

**Keywords:** *Arachis hypogaea*, Calmodulin/calmodulin-like proteins, *Ralstonia solanacearum*, Genome-wide, Resistance

## Abstract

**Background:**

Calmodulins (*CaMs*)/CaM-like proteins (CMLs) are crucial Ca^2+^-binding sensors that can decode and transduce Ca^2+^ signals during plant development and in response to various stimuli. The *CaM/CML* gene family has been characterized in many plant species, but this family has not yet been characterized and analyzed in peanut, especially for its functions in response to *Ralstonia solanacearum*. In this study, we performed a genome-wide analysis to analyze the *CaM/CML* genes and their functions in resistance to *R. solanacearum*.

**Results:**

Here, 67, 72, and 214 *CaM/CML* genes were identified from *Arachis duranensis*, *Arachis ipaensis*, and *Arachis hypogaea*, respectively. The genes were divided into nine subgroups (Groups I-IX) with relatively conserved exon‒intron structures and motif compositions. Gene duplication, which included whole-genome duplication, tandem repeats, scattered repeats, and unconnected repeats, produced approximately 81 pairs of homologous genes in the *AhCaM/CML* gene family. Allopolyploidization was the main reason for the greater number of *AhCaM/CML* members. The nonsynonymous (Ka) versus synonymous (Ks) substitution rates (less than 1.0) suggested that all homologous pairs underwent intensive purifying selection pressure during evolution. *AhCML69* was constitutively expressed in different tissues of peanut plants and was involved in the response to *R. solanacearum* infection. The AhCML69 protein was localized in the cytoplasm and nucleus. Transient overexpression of *AhCML69* in tobacco leaves increased resistance to *R. solanacearum* infection and induced the expression of defense-related genes, suggesting that *AhCML69* is a positive regulator of disease resistance.

**Conclusions:**

This study provides the first comprehensive analysis of the *AhCaM/CML* gene family and potential genetic resources for the molecular design and breeding of peanut bacterial wilt resistance.

**Supplementary Information:**

The online version contains supplementary material available at 10.1186/s12864-024-10108-5.

## Background

Plants are faced with various stresses throughout their lifetime. To overcome these challenges, plants perceive and translate these external stimuli into an internal response via complex signaling networks. Calcium (Ca^2+^), a second messenger of signal transduction in plant cells, plays important roles in plant growth and development as well as in coping with stress [1]. When plants experience external stimulation, the concentration of cytosolic free Ca^2+^ increases, which activates a calcium signature [2]. These Ca^2+^ signatures are decoded and transmitted into downstream responses by a toolkit of calcium-binding proteins, which are referred to as calcium sensors [3]. The major sensors, e.g., calmodulins (CaMs), CaM-like proteins (CMLs), Ca^2+^-dependent protein kinases (CDPKs), and calcineurin B-like proteins (CBLs), usually contain a number of EF-hand motifs [4, 5]. Each EF-hand motif consists of 29 amino acid residues forming two α-helices that are connected by a 12-amino acid loop [6]. The Asp (D) and Glu (E) amino acids in the EF-hand motif are conserved and constitute the D-x-D or D/E-E-L motif, which can bind to Ca^2+^ [7, 8]. An EF-hand binds one Ca^2+^ ion, inducing a conformational change in the Ca^2+^ sensor protein, interacting with downstream proteins or regulating their catalytic activity [9, 10].

*CaMs*, which have four EF-hand motifs, are conserved Ca^2+^ sensors in both plants and animals. *CMLs*, normally with 1–6 EF-hand motifs, display some sequence homology with *CaM* and exhibit structural differentiation in plants [11, 12]. Genome-wide analysis of *CaM/CML* genes has been performed for many plant species, including *Arabidopsis* (7 *CaMs* and 50 *CMLs*), rice (*Oryza sativa*, 5 *CaMs* and 32 *CMLs*), *Brassica napus* (25 *CaMs* and 168 *CMLs*), tomato (*Solanum lycopersicum*, 6 *CaMs* and 52 *CMLs*), and wheat (*Triticum aestivum*, 18 *CaMs* and 230 *CMLs*) [13, 14]. Although *CMLs* are structurally homologous to *CaMs*, plants have far more *CMLs* than *CaMs*. *CaM/CMLs* are widely involved in plant growth and development, as well as in the response to various environmental stimuli [15]. In *Arabidopsis*, *CaM3* activates the shock transcription factor (HSF) by regulating the activity of CaM-binding protein phosphatase (PP7) or protein kinase (CBK3), resulting in heat resistance [16, 17]. CMLs have diverse functions in the plant immune response, and *AtCML8*, *AtCML9*, and *AtCML24* function as positive regulators in response to *Pseudomonas syringae pv. Tomato DC3000*, while *AtCML46* and *AtCML47* have been demonstrated to be negative monitors [18, 19]. *CMLs* can be regulated by transcription factors (MYB and bZIP) or miRNAs to form a feedback loop to confer pathogen resistance. In upland cotton, GhMYB108 interacts with *GhCML11* and forms a positive feedback regulatory loop to participate in resistance to *Verticillium dahlia* infection [20]. Pepper *CaCML13* is positively regulated by CabZIP63 at the transcriptional level and forms a positive feedback network to prevent *R. solanacearum* infection (RSI) [21]. In rice, overexpression of *OsCaML2* reduces resistance to *Xanthomonas oryzae pv. Oryzae (Xoo)* [22]. *OsCaML2* was demonstrated to be the target protein of *osa-miR1432*, and the highly inducible *osa-miR1432* suppressed the expression of *OsCaML2* to increase disease resistance. The *CML*-mediated network can induce changes in downstream hormone levels, including changes in salicylic acid (SA) and jasmonic acid (JA) signaling. In tomato, silencing *SlCML55* increased *PR1* expression and stimulated the SA immune response, ultimately resulting in resistance to *Phytophthora capsica* [23]. *Arabidopsis AtCML37* and *AtCML42* have been demonstrated to play roles in defense against herbivory and some pathogen attacks, which is related to calcium and JA signaling [24–26]. Although the function of *CaMs/CMLs* in response to various stimuli has been systematically studied in several plant species, genome-wide analysis of the *CaM/CML* gene families of peanut has rarely been performed, and the possible functions of peanut *CaM/CML* genes are still unclear.

Cultivated peanut is an important oil and economic crop in tropical and subtropical regions of the world. Bacterial wilt (BW) is a destructive soilborne disease caused by *R. solanacearum* that can cause severe problems in peanut yield and quality. More than 200 plant species from 54 families can be infected by *R. solanacearum*, resulting in very serious economic losses worldwide every year [27]. Such losses may cause more than 10% decreases in peanut production or even result in the death of the entire crop. Although many disease resistance (*R*) genes and resistance-related genes have been found in plants, the *R* genes for BW are still poorly understood [28]. Two *R* genes, *RRS1-R/RPS4* and *ERECTA*, act as positive regulators of resistance to BW and have been extensively studied in *A. thaliana* [29]. Only three BW resistance-related genes, *AhRLK1*, *AhRRS5*, and *AhGLK1b*, have been cloned from peanut [30–32]. Separately overexpressing the three genes in tobacco caused resistance to *R. solanacearum*. As a result, additional studies are necessary to identify BW resistance (or related) genes in peanut plants, which will be beneficial for revealing the molecular pathways involved in peanut resistance to *R. solanacearum* and accelerating the breeding of peanut plants resistant to BW.

In the present study, we first performed a genome-wide analysis to identify *CaM/CML* genes in *A. duranensis*, *A. ipaensis* and *A. hypogaea*. The characteristics of the *CaM/CML* genes, including intron‒exon organization, chromosomal location, EF-hand motifs, and phylogenetic relationships, were evaluated. To demonstrate the involvement of the *CaM/CML* genes in peanut responses to *R. solanacearum*, the expression patterns of these genes were also analyzed through RNA-seq data from peanut leaves with RSI [33]. The function of *AhCML69* was further investigated due to its significantly upregulated expression following *R. solanacearum* infection. Transient overexpression of *AhCML69* increased the resistance to *R. solanacearum* in *Nicotiana benthamiana*. Our results will be useful for understanding the functions of *AhCaM/CML* in modulating peanut responses to *R. solanacearum*.

## Results

### Identification and characterization of CaM/CML genes

Through a BLASTP search of *Arabidopsis AtCaM/CML* protein sequences, 67, 72, and 214 *CaM/CML* genes were identified in *A. duranensis* (*AdCaMs/CMLs*), *A. ipaensis* (*AiCaMs/CMLs*), and *A. hypogaea* (allotetraploid peanut cultivar; *AhCaMs/CMLs*), respectively. All the members were submitted to InterPro and SMART to verify the presence of the EF-hand motif domain. All the *CaM* proteins and 70 *AhCML*, 13 *AdCML* and 10 *AiCML* proteins had four EF-hand motifs. The remaining *CML* proteins had varying numbers of EF-hand motifs (1, 2, 3, 4 or 6) (Additional File 1). Detailed information for all the identified *CaM/CML* genes, including gene ID, chromosome location, molecular weight (MW), isoelectric point (PI), and number of EF-hand motifs, is provided in Additional File 1.

### Chromosome distribution and gene structure analysis of the CaM/CML genes

Chromosomal location analysis revealed that these *CaM/CML* genes were unevenly distributed among the chromosomes (Fig. S1A-C). In peanut, chromosome 13 had the highest number of *AhCaM/CML* genes, containing 19 (8.87%), while chromosomes 9, 10, 19 and 20 had the lowest number of *AhCaM/CML* genes, each containing 5 (2.33%) (Fig. S1C and Additional File 1). The *AdCaM/CML* genes were unevenly distributed across ten chromosomes of *A. duranensis*, with a maximum of 10 (14.93%) on chromosome 6 and only 3 (4.48%) on chromosome 10 (Fig. S1A and Additional File 1). Similarly, the *AiCaM/CML* genes were unevenly distributed across ten chromosomes of *A. ipaensis*. Chromosomes 2 and 6 had the highest number of *AiCaM/CML* genes (12; 16.4%), while chromosome 10 had the lowest number of *AiCaM/CML* genes (3; 4.1%) (Fig. S1B). All the *CaM/CML* genes were renamed according to their chromosomal location (Additional File 1).

The gene structures of the *CaM/CML* members were analyzed according to their exon‒intron organization. Most of the *CaM/CML* genes had more than one intron (Fig. S1D-F). No introns were found in 77 (35.98%) *AhCaM/CML* genes, while more than one intron was identified in the other genes (Fig. S1F). In the two diploid species, the *CaM/CML* gene structures were generally similar. There were 12 (17.65%) *AdCaM/CML* genes with no introns, and the others contained 3-5 introns in *A. duranensis* (Fig. S1D). The number of introns varied from 0 to 11 in *A. ipaensis*. There were 14 (19.44%) *AiCaM/CML* genes without introns, and the others had 1-11 introns (Fig. S1E).

### Phylogenetic and conserved motifs of CaM/CML proteins

**Fig. 1:**
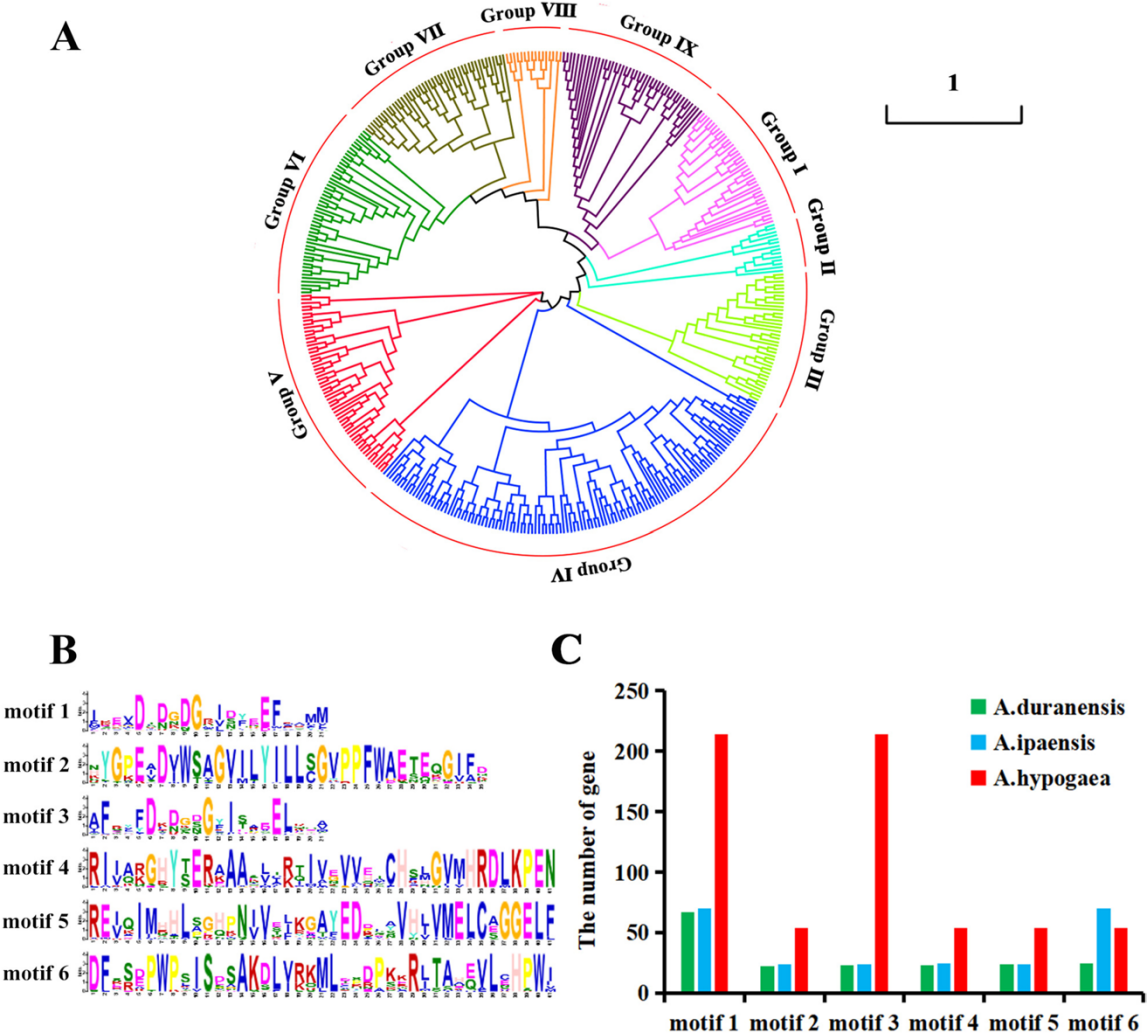
Phylogenetic and conserved motif analysis of the CaM/CML proteins in *A. hypogaea*, *A. duranensis* and *A. ipaensis.* **A** Phylogenetic tree of CaM/CML proteins from *A. hypogaea*, *A. duranensis*, *A. ipaensis*, and *Arabidopsis* was constructed with 1000 bootstrap replications*.* The different subgroups are distinguished using different colors. **B** Sequence logo of the six motifs. **C** The distributions of motifs 1-6 in the AhCaM/CML*,* AdCaM/CML, and AiCaM/CML proteins

To evaluate the evolutionary relationships of the *CaM/CML* gene family, we constructed a phylogenetic tree by using the NJ method (1000 bootstrap replicates) to associate the AdCaM/CML, AiCaM/CML, and AhCaM/CML proteins with the *Arabidopsis* AtCaM/CML proteins. These CaM/CML proteins were divided into nine subgroups (Fig. 1A and Additional File 3). Group I included 37 members (7 AtCaMs, 3 AdCaMs, 4 AiCaMs and 23 AhCaMs); Group II included 17 CaM members (7 AdCaMs and 10 AiCaMs); All CaM members were divided into Group I and Group II; Group III contained 37 CML members (9 AtCMLs, 4 AdCaMs, 10 AiCaMs and 14 AhCaMs); and Group IV included 165 CML members (5 AtCMLs*,* 24 AdCaMs, 23 AiCaMs and 113 AhCaMs), with most CMLs in subgroup IV (164 CMLs). Groups V, VI, VII, VIII and IX included 34, 33, 32, 16, and 39 members, respectively. The smallest subgroup was VIII, which consisted of sixteen CMLs with no AhCMLs. Notably, in subgroup V, several AhCMLs monopolized a small branch, which might indicate specialized functions.

By analyzing the domains and motifs of the identified CaM/CML proteins, six additional motifs were predicted via the MEME website (Fig. 1B and Additional File 4). Motif 1 includes the core conserved sequence (D-x-D) in the EF-hand motif, and motif 2 contains a helix-loop-helix sequence. All CaM/CML proteins consist of one to six copies of motif 1. All Group I, II (CaMs) and IV proteins had four copies of motif 1. The other group of CML proteins included motifs 2-5 (Fig. 1C and Additional Files 3 and 4). Sequence analysis of AhCaM/CML proteins demonstrated that both AhCaMs and AhCMLs contain EF-hand motifs, while the sequence similarity of CaMs with EF-hand motifs was greater than that of *CMLs.*

### Gene duplication and collinearity analysis of the CaM/CML genes

**Fig. 2:**
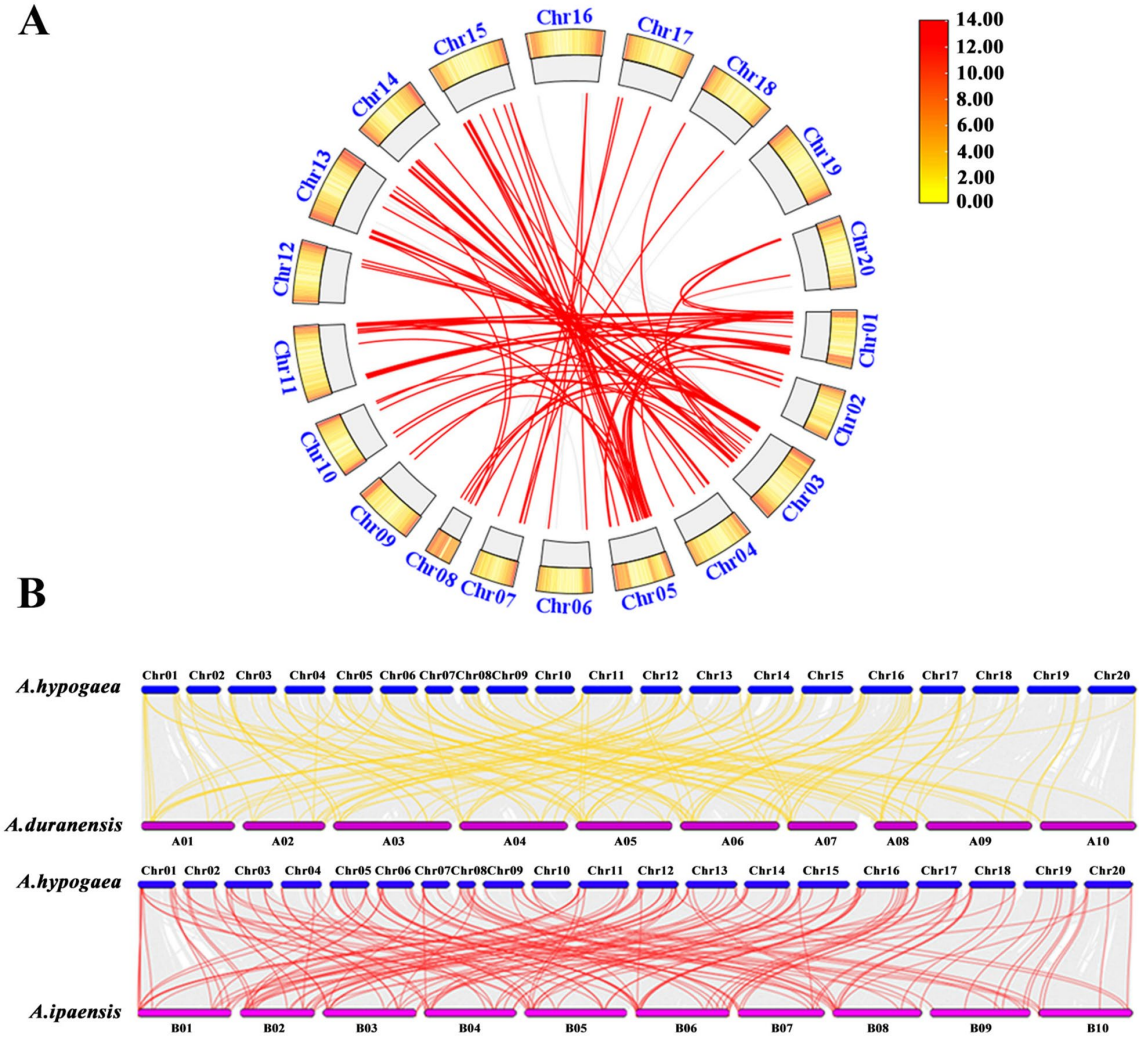
Collinearity analysis of *CaM/CML* genes in *A. hypogaea*, *A. duranensis* and *A. ipaensis*. **A** Duplication events in *AhCaM/CML* genes. The red lines indicate collinear *CaM/CML* gene pairs in *A. hypogaea*. **B** Collinearity analysis of the *CaM/CML* genes in *A. hypogaea*, *A. duranensis* and *A. ipaensis*. Homologous *CaM/CML* gene pairs between species are linked by yellow and red lines, respectively

To elucidate the role of gene duplication in the evolution of the *AhCaM/CML* genes, the duplication events of these genes were investigated via collinearity analysis. A total of 81 pairs of homologous *AhCaM/CML* genes were found in peanut (Fig. 2A and Additional File 5). Further analysis revealed that 214 *AhCaM/CML* genes were derived from gene duplication, of which 110 genes were derived from whole-genome duplication, 71 from tandem repeats, and 33 from scattered repeats (Additional File 6). These findings indicated that the expansion of the *AhCaM/CMLs* occurred mainly through genome-wide duplication. In addition, we investigated the synteny relationships of the *CaM/CML* genes between peanut and the two wild species. Sixty-seven pairs of homologous *CaM/CML* genes were identified between peanut and *A. duranensis*, and 72 pairs of homologous *CaM/CML* genes were identified between peanut and *A. ipaensis* (Fig. 2B and Additional File 7). These results suggested that the allotetraploid peanut evolved from these two wild species. To explore the evolutionary selection pressure on *AhCaM/CML* genes, the Ka/Ks values of the *AhCaM/CML* gene pairs were calculated using TBtools software. The results showed that the Ka/Ks ratios of all ortholog pairs were less than 1.0 (Additional File 8), indicating that the homologous genes had undergone intensive purifying selection pressure and remained conserved in both structure and function in peanut.

### Expression profile analysis of AhCaM/CML genes in response to R. solanacearum

**Fig. 3:**
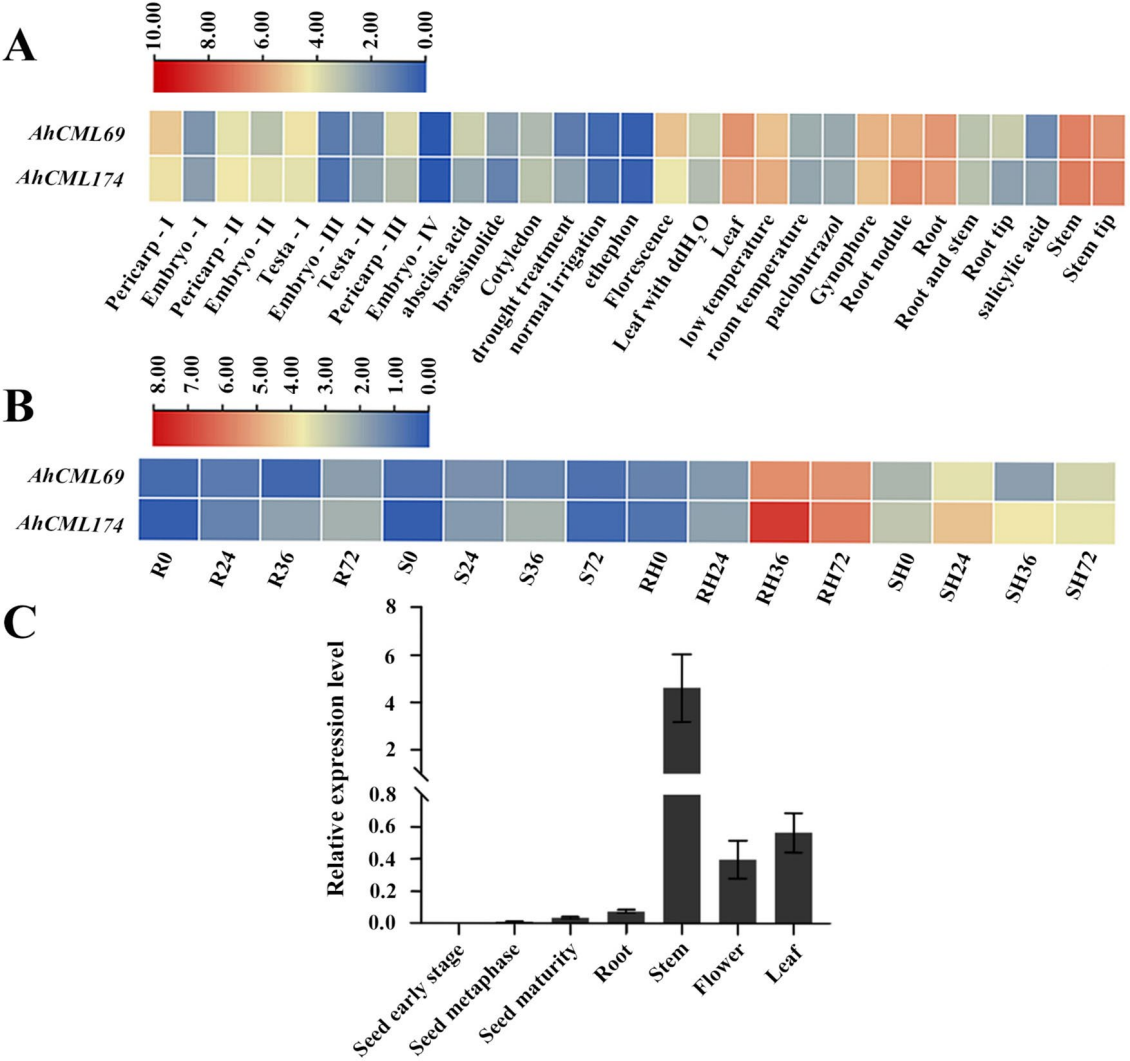
Heatmap of the expression profiles of the *AhCaM/CML* genes and expression analysis of *AhCML69*. **A** Expression profiles (in log10-based FPKM) of the representative *AhCaM/CML* genes from 29 peanut tissues. The expression abundance of each gene is represented by the color bar: red indicates higher expression, and green indicates lower expression. FPKM, Fragments Per Kilobase of Transcript per Million mapped reads. **B** Expression profiles (in log10-based FPKM) of the representative *AhCaM/CML* genes in the leaves of resistant and susceptible peanut plants at 0, 24, 36, 48 and 72 h post-inoculation with *R. solanacearum*. **C** Expression analysis of *AhCML69* in different peanut tissues. Error bars represent standard error, n=3

To explore the potential functions of the *AhCaM/CML* genes in response to *R. solanacearum* infection, we analyzed the expression patterns of these genes in resistant and susceptible peanuts after *R. solanacearum* infection based on previous transcriptome data [33]. Two *AhCML* genes, *AhCML69* (*AH08G06260.1*) and *AhCML174* (*AH17G29650.1*), were selected as candidate genes due to their high expression after *R. solanacearum* infection (Fig. 3B). The spatial and temporal expression profiles of the two *AhCML* genes were further investigated based on publicly available transcriptome datasets [34]. These genes were highly expressed in the roots, stems, leaves, and flowers (Fig. 3A), indicating that they may have extensive functions. To verify the consistency of the expression pattern, the expression of *AhCML69* in different tissues of peanut plants was determined via qRT‒PCR in three biological replicates. These results were in substantial agreement with the previous transcriptome analysis results, which revealed that the gene was highly expressed in the roots, stems, leaves, and flowers (Fig. 3C). In addition, the expression of *AhCML69* and *AhCML174* increased in response to low temperature or salicylic acid treatment (Fig. 3A), suggesting that these genes are likely involved in the response to environmental stimuli.

### Cloning and functional validation of candidate AhCML genes

**Fig. 4:**
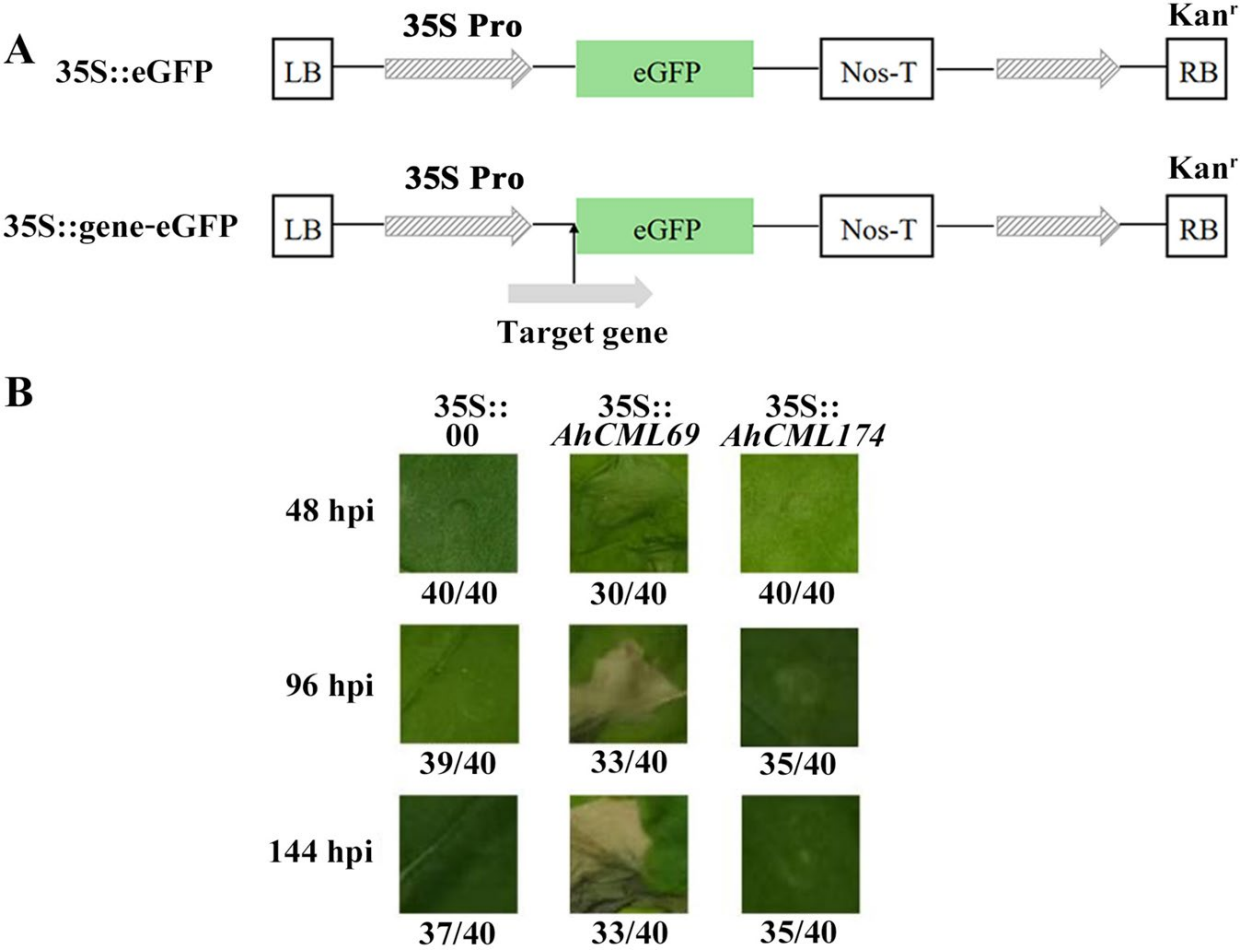
Functional analysis of the representative *AhCML* genes. **A** Structure of the transient overexpression vectors containing *AhCML69* and *AhCML174.* **B** Phenotypic analysis of tobacco leaves agroinfiltrated with *Agrobacterium* in the control (*35S::00*) and experimental (*35S::AhCML69* and *35S::AhCML174*) groups at 48, 72, 96, 120 and 144 h. The numbers indicate the number of agroinfiltrated leaves on the bottom

The two *AhCML* genes *AhCML69* and *AhCML174* were successfully cloned from the leaf cDNA of resistant peanut plants infected with *R. solanacearum*. The ORF lengths were 488 and 908 bp, encoding 163 and 303 amino acids, respectively (Additional File 9). To analyze the functions of these genes in plants, transient overexpression vectors encoding *AhCML69* and *AhCML174* were constructed and subsequently transformed into the leaves of *N. benthamiana* via *Agrobacterium* transformation (Fig. 4A). Compared with that in control leaves agroinfiltrated with *35S::00*, leaf necrosis was obvious at 48 hours post-agroinfiltration in the *AhCML69-* and *AhCML174*-overexpressing leaves, although necrosis was less evident in the *AhCML174*-overexpressing leaves (Fig. 4B). These phenotypic results demonstrated that *AhCML69* and *AhCML174* might play a certain role in mediating cell death to varying degrees.

### Sequence analysis and subcellular localization of the AhCML69 protein

**Fig. 5:**
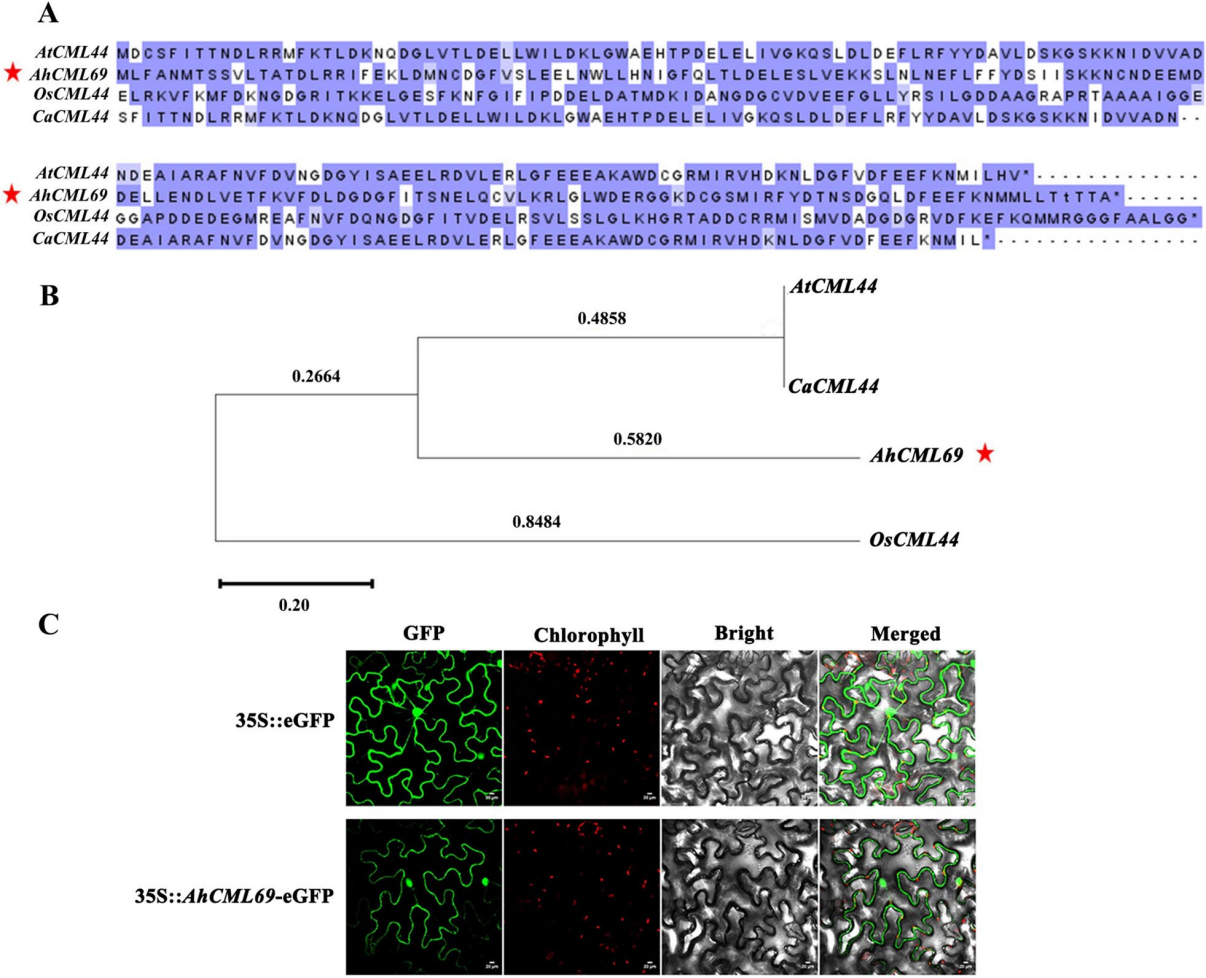
Sequence analysis and subcellular localization of AhCML69. **A** Multiple sequence alignment of the CML44 proteins from *Arabidopsis*, rice*,* pepper, and *A. hypogaea*. **B** Phylogenetic tree of the CML44 proteins in *Arabidopsis*, rice*,* pepper, and *A. hypogaea*. **C** Subcellular localization of the AhCML69 protein

The *AhCML69* gene encodes a calmodulin-like protein with four EF-hand motifs and is located on chromosome 8 (Additional File 1). This protein consists of 163 amino acids; and has a MW of 18746.22 and a pI of 4.17 (Additional File 1). To further evaluate the sequence similarity between *AhCML69* and other *CMLs*, BLASTP analysis was performed by using the full-length amino acid sequence from the NCBI website, and the results suggested that *AhCML69* was closely related to *Arabidopsis AtCML44*, rice *OsCML44* and pepper *CaCML44*. Multiple sequence alignment and phylogenetic analysis also verified that AhCML69 was highly similar to AtCML44, OsCML44, and CaCML44 (Fig. 5A, B).

To determine the subcellular localization of AhCML69, the recombinant plasmid PC2300-35S-*AhCML69*-eGFP (35S::*AhCML69*::eGFP) and the control PC2300-35S-eGFP (*35S::eGFP*) were transformed into *N. benthamiana* leaves by *Agrobacterium*-mediated transient expression. The infiltrated leaves were cut and observed under a confocal laser microscope at 48 hours post-infiltration (hpi). Compared with the control (*35S::eGFP*), the AhCML69 protein (35S::*AhCML69*::eGFP) emitted green fluorescence in the cytoplasm and nucleus (Fig. 5C), indicating that the fusion protein AhCML69::eGFP was localized in the cytoplasm and nucleus.

### Transient overexpression of AhCML69 causes cell death in N. benthamiana leaves

**Fig. 6:**
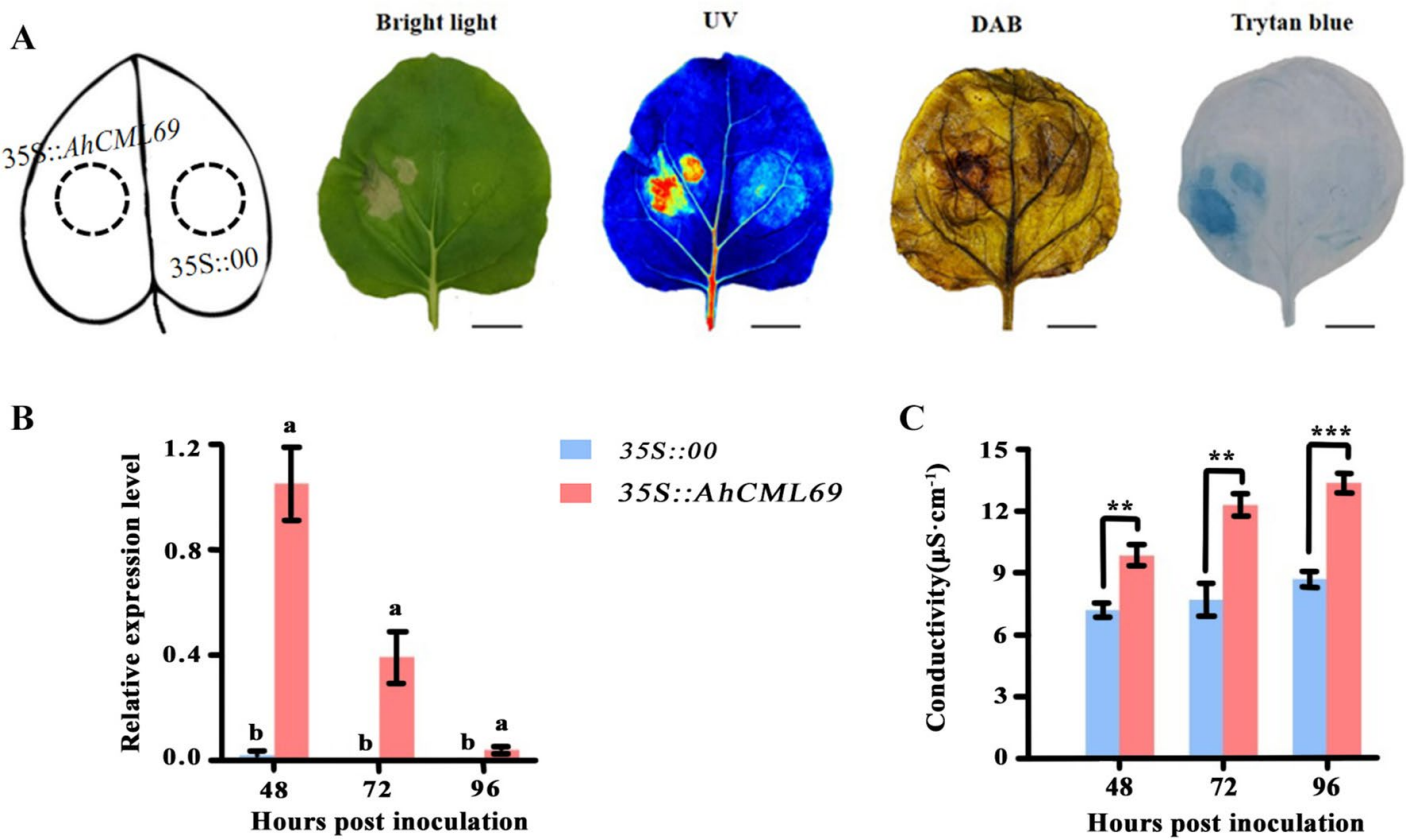
Analysis of the function of *AhCML69* in *N. benthamiana* leaves. **A** Phenotypic, trypan blue and DAB staining analyses of the tobacco leaves agroinfiltrated with *Agrobacterium* and the control (*35S::00*) or experimental (*35S::AhCML69*) groups at 48 h. Cell death was monitored under visible light (Camera) and UV light (UV); bar = 5 cm. **B** The expression levels of *AhCML69* in tobacco leaves agroinfiltrated with *Agrobacterium* containing *35S::00* or *35S::AhCML69* at 48, 72 and 96 h. Different lowercase letters indicate significant differences according to ANOVA (means ± SEs, *p* < 0.05). **C**. Electrolyte leakage in tobacco leaves agroinfiltrated with *Agrobacterium* containing *35S::00* and *35S::AhCML69* at 48, 72 and 96 h. The asterisks indicate significant differences between *35S::00* and *35S::AhCML69* according to Student*’*s t test (mean ± SE, *p < 0.05, **p < 0.001, ***p < 0.0001)

To further demonstrate that *AhCML69* is involved in disease resistance, the recombinant plasmid *35S::AhCML69* and the control *35S::00* were transiently overexpressed in *N. benthamiana* leaves by *Agrobacterium* transformation. *AhCML69* was highly expressed at 48, 72, and 96 hpi in the *35S::AhCML69* leaves of *N. benthamiana* (Fig. 6B). Compared with that in *35S::00* plants, cell death was more severe in the leaves of *35S::AhCML69* plants at 48 hpi, which was also verified by trypan blue staining and electrolyte leakage measurements (Fig. 6AC). In the trypan blue staining experiment, the color of the *35S::AhCML69* leaves was deeper blue than that of the control leaves at 48 hpi, and the electrolyte leakage of the *35S::AhCML69* leaves was significantly greater than that of the control leaves at 48, 72, and 96 hpi. These results suggested that there was severe cell death in the leaves of *35S::AhCML69*. DAB (3,3'-diaminobenzidine) staining revealed significantly greater brown staining in the *35S::AhCML69*-overexpressing leaves than in the control leaves at 48 hpi, indicating that *AhCML69* increased the content of active oxygen species and was likely involved in the plant defense response.

### Transient overexpression of AhCML69 positively mediates the defense response

**Fig. 7:**
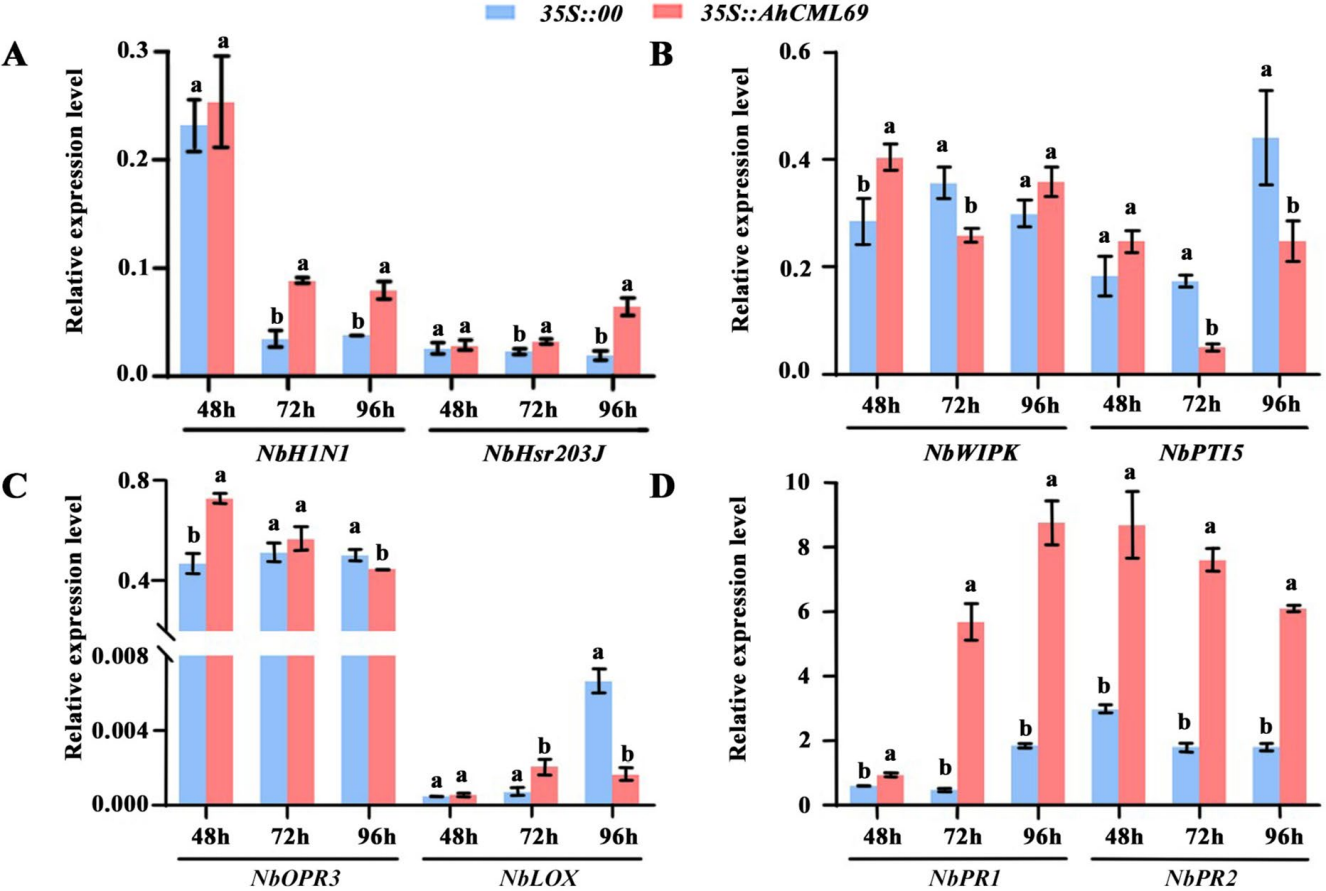
Expression analysis of immune marker genes in tobacco leaves agroinfiltrated with *35S::00* and *35S::AhCML69* at 48, 72 and 96 h. HR marker genes: *NbH1N1* and *NbHsr203J*; PTI marker genes: *NbWIPK* and *NbPTI5*; JA signaling pathway genes: *NbOPR3* and *NbLOX;* SA signaling pathway genes: *NbPR1* and *NbPR2*. Different lowercase letters indicate significant differences according to ANOVA (mean ± SE, *p* < 0.05)

To confirm the resistance effect of *AhCML69*, the expression levels of immune-related marker genes were determined in *35S::AhCML69* and *35S::00* leaves at 48, 72 and 96 hpi by qRT‒PCR (Fig. 7). Compared with those in *35S::00* leaves, in *35S::AhCML69* leaves, the expression of the hypersensitive response (HR) marker genes *NbH1N1* and *NbHsr203J* was significantly upregulated at 72 and 96 hpi, indicating that cell death was associated with HR (Fig. 7A). The PTI (PAMP-triggered immunity)-related genes *NbWIPK* at 48 and 96 hpi and *NbPTI5* at 48 hpi were upregulated in *35S::AhCML69* leaves (Fig. 7B). These results suggested that AhCML69 positively regulates the PTI response. The JA signaling pathway-related genes *NbOPR3* at 48 hpi and *NbLOX* at 72 hpi and the SA signaling pathway-related genes *NbPR1* and *NbPR2* at all timepoints were upregulated in *35S::AhCML69* leaves (Fig. 7C, D). These results suggested that *AhCML69* positively regulates the defense response by regulating the JA and SA signaling pathways.

### Transient overexpression of AhCML69 enhanced resistance to R. solanacearum

**Fig. 8:**
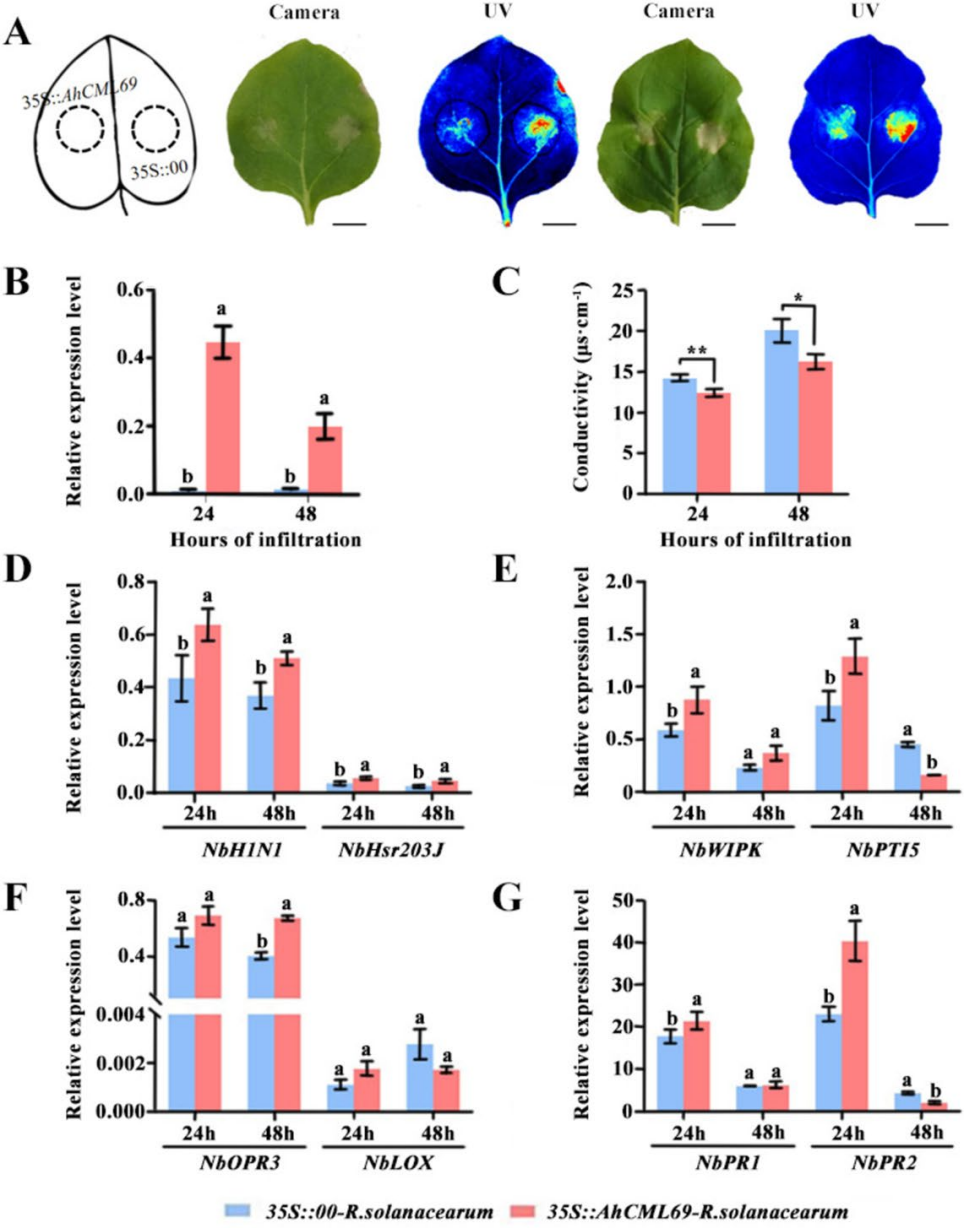
Analysis of the function of *AhCML69* in resistance to *R. solanacearum* in *N. benthamiana* leaves. **A** Phenotypic analysis of the tobacco leaves after inoculation with *R. solanacearum* for 24 and 48 h (from left to right). Cell death was monitored by visible light (Camera) and UV light (UV); bar = 5 cm. **B** The expression levels of *AhCML69* in tobacco leaves agroinfiltrated with *Agrobacterium* containing *35S::00* or *35S::AhCML69* at 24 and 48 h. Different lowercase letters indicate significant differences according to ANOVA (mean ± SE, *p* < 0.05). **C** Electrolyte leakage in tobacco leaves agroinfiltrated with *Agrobacterium* expressing *35S::00* or *35S::AhCML69*. Different lowercase letters indicate significant differences according to ANOVA (mean ± SE, *p* < 0.05). **D**-**G** Expression analysis of immune marker genes in tobacco leaves agroinfiltrated with *35S::00* and *35S::AhCML69* at 48, 72 and 96 h. Different lowercase letters indicate significant differences according to ANOVA (means ± SEs, *p* < 0.05)

To determine the function of *AhCML69* in resistance to *R. solanacearum*, we transiently overexpressed *35S::AhCML69* and *35S::00* in *N. benthamiana* leaves and subsequently inoculated them with *R. solanacearum*. qRT‒PCR in *35S::AhCML69* tobacco leaves at 24 and 48 hpi revealed high expression of *AhCML69* (Fig. 8B), indicating its successful expression. Disease symptoms and the expression levels of immune-related marker genes were subsequently evaluated in tobacco leaves. Compared with that in *35S::00* plants, cell death in *35S::AhCML69* tobacco leaves was lower after *R. solanacearum* inoculation (Fig. 8A), which was also further demonstrated by electrolyte leakage measurements (Fig. 8C). These findings suggested that cell death, a disease symptom caused by *R. solanacearum* infection, was retarded to a certain degree. The HR marker genes *NbH1N1* and *NbHsr203J* at 24 and 48 hours post-inoculation with *R. solanacearum* (hpir), the PTI-related gene *NbWIPK* at 24 and 48 hpir, and *NbPTI5* at 24 hpir were upregulated in *35S::AhCML69* leaves (Fig. 8D, E). The JA signaling pathway-related genes *NbOPR3* at 24 and 48 hpir and *NbLOX* at 24 hpir and the SA signaling pathway-related genes *NbPR1* and *NbPR2* at 24 hpir were upregulated in *35S::AhCML69* leaves (Fig. 8F, G). These results suggested that *AhCML69* enhances tobacco resistance to *R. solanacearum* by regulating the JA- and SA-associated PTI signaling pathways.

## Discussion

CaM is a conserved protein found in both plants and animals, while CML is a unique protein found in plants. The *CaM/CML* gene family has been demonstrated to play important roles in plant development and the response to various stimuli [15]. However, there have been no genome-wide analyses of the *CaM/CML* gene family in *Arachis* species. In the present study, the distribution and structural and functional characteristics of the *CaM/CML* genes in *A. hypogaea* were investigated, which could provide a comprehensive understanding of the evolutionary history and functional roles of this gene family. Twenty-three *CaM* and 191 *CML* members were identified in peanut, which is more than in most plant species, including *A. duranensis* (9 *CaM* and 58 *CML*), *A. ipaensis* (14 *CaM* and 58 *CML*), *Arabidopsis* (7 *CaMs* and 50 *CMLs*), rice (5 *CaMs* and 32 *CMLs*), tomato (6 *CaMs* and 52 *CMLs*), and *B. napus* (25 *CaMs* and 168 *CMLs*) [14]. This is likely because peanut (AABB, 2n = 40) was formed by natural allopolyploidization between *A. duranensis* (AA, 2n = 20) and *A. ipaensis* (BB, 2n = 20), and its genome (2.38 Gb) is larger than that of the other plants. Collinearity analysis revealed that almost all the *CaM/CML* genes in the two wild species were homologous to genes in peanut (Fig. 2B and Additional File 7), indicating that the allotetraploid peanut evolved from these two diploid species. Gene duplication, often derived from polyploidization (mainly segmental, whole-genome or tandem duplication), plays important roles in gene family expansion [35, 36]. There were 81 pairs of homologous of *CaM/CML* genes in peanut (Fig. 2A and Additional File 5). Among the *AhCaM/CML* genes, whole-genome duplication, tandem repeats, and scattered repeats were the most common duplication types (Additional File 6). To illustrate the evolutionary limitations and selection pressures on the *AhCaM/CML* genes, Ka, Ks, and Ka/Ks values for 81 homologous *AhCaM/CML* gene pairs were calculated (Additional File 8). Ks values between gene pairs, indicating the rate of background base substitution, can be used to estimate the time since whole-genome duplication [37]. The Ks values for the *AhCaM/CML* gene pairs varied from 0.007 to 2.08, suggesting that a large-scale *AhCaM/CML* gene duplication event occurred (Additional File 8). The Ka/Ks ratios of all ortholog pairs were less than 1.0 in peanut (Additional File 8), suggesting that the homologous *AhCaM/CMLs* were subjected to intensive purifying selection pressure and remained conserved in both structure and function during evolution.

The spatiotemporal expression patterns of the *AhCaM/CML* genes may be associated with their functions. Our previous study showed that many Ca^2+^ sensor genes are significantly induced by RSI in peanut leaves [33]. The potential relationship between the expression levels of the *AhCaM/CML* genes and RIS was investigated, and the expression levels of *AhCML69* and *AhCML174* were found to be positively correlated with RIS in peanut leaves (Fig. 3B). Tissue expression analysis demonstrated that *AhCML69* and *AhCML174* were highly expressed in roots, stems, leaves, and flowers (Fig. 3AC). In addition, *AhCML69* and *AhCML174* were induced by low-temperature or salicylic acid treatment (Fig. 3A). In *Arabidopsis*, *AtCML8* is highly expressed in roots, leaves, and flowers and is involved in resistance to *R. solanacearum* [38]. *CaCML13* is significantly upregulated by RIS in pepper roots, where *R. solanacearum* invades, and plays an important role in pepper immunity against *R. solanacearum* [21]. *TaCML36* is significantly induced by the soil-borne fungus *Rhizoctonia cerealis* in resistant wheat stems where disease symptoms appear and positively participates in the immune response to *R. cerealis* [39]. Plant roots, especially the lateral roots and tips, are the main sites where *R. solanacearum* invades the host [40]. Once *R. solanacearum* enters plant roots, it invades xylem vessels to spread toward the aerial parts of the host through the vascular system, ultimately resulting in BW [41, 42]. *AhCML69* was highly expressed in the roots, stems, and leaves, indicating that it participated in the entire process of resistance to *R. solanacearum* in peanut plants. Subcellular localization assays indicated that the *AhCML69* protein was localized in both the cytoplasm and nucleus (Fig. 5C). Several *CML* proteins, such as *NbCML30*, *GhCML11*, *TaCML36*, and *CaCML13*, have likewise been demonstrated to be located in both the cytoplasm and nucleus and to be involved in the response to biotic stress [15]. Silencing of *NbCML30* increased tobacco mosaic virus (TMV) infection, while its overexpression inhibited TMV invasion [43]. GhCML11 interacts with GhMYB108 to form a positive feedback loop to enhance the defense response against *Verticillium dahliae* infection in upland cotton [20]. Its nuclear-cytoplasmic localization suggested that *AhCML69* possibly functions by activating proteins from the nucleus and cytoplasm [44]. These results suggested that *AhCML69* is involved in the response to pathogens.

As an important oil crop and protein source, peanut plants can be grown in poor-quality soil [45, 46], but their yield is significantly restricted by BW caused by *R. solanacearum* [27]. However, studies on the mechanisms of the peanut-*R. solanacearum* interaction are rare, and no *R* genes conferring resistance to BW have been identified in peanut. During plant‒pathogen interactions, plants activate the two-layer innate immune system, pattern-triggered immunity (PTI) and effector-triggered immunity (ETI), which cause a series of early signaling events, including Ca^2+^ flux, mitogen-activated protein kinase (MAPK) activation, reactive oxygen species (ROS) production, the induction of plant hormone biosynthesis, and the hypersensitive response (HR) [47–49]. As the earliest event, the influx of Ca^2+^ from outside plant cells is essential for the immune response [50, 51]. Ca^2+^ signaling is decoded and transduced by sensors that include *CML* proteins. Accumulating evidence has demonstrated that *CMLs* are involved in plant protection against pathogen infection [15]. In the present study, compared with those in *35S::00* tobacco leaves, leaf necrosis and ROS accumulation were obvious in *35S::AhCML69* tobacco leaves at 72 hpi (Fig. 6A). These findings were consistent with those of two other resistance-related genes identified in previous studies, in which overexpression of *AhRRS5* or *AhRLK1* in *N. benthamiana* leaves induced HR and cell death [30, 31]. Moreover, after *35S::AhCML69* tobacco leaves were inoculated with *R. solanacearum*, the degree of leaf necrosis was significantly less severe than that in *35S::00* leaves (Fig. 8A). These results demonstrated that the transient overexpression of *AhCML69* in tobacco increased its resistance to *R. solanacearum* infection.

Evidence indicates that *CMLs* are involved in modulating the transcription of defense-related genes, ultimately causing plant resistance against pathogen infection. As downstream signaling molecules, SA and JA are known plant hormones associated with plant‒pathogen interactions [52]. *AtCML8* and *AtCML9* are induced by SA treatment and increase the expression of the SA-dependent *PR1* gene in *Arabidopsis* in response to *P. syringae* [19, 53]. *AtCML37* and *AtCML42* are involved in the defense against herbivory and some pathogen attacks and are associated with calcium and JA signaling [24]. In this study, compared with those in the *35S::00* control, the expression of the SA signaling marker genes *NbPR1* and *NbPR2* and the JA signaling gene *NbOPR3* and *NbLOX* was significantly upregulated in *35S::AhCML69* tobacco leaves infected with *R. solanacearum* (Fig. 8F, G). Moreover, the expression of the HR marker genes *NbH1N1* and *NbHsr203J* and the PTI marker genes *NbWIPK* and *NbPTI5* was also significantly induced after inoculation of *35S::AhCML69* tobacco leaves with *R. solanacearum* (Fig. 8D, E). These results were consistent with those elucidated in the study of *AhRRS5* and *AhRLK1* function, where HR-, JA- and SA-signaling pathway-related genes were all induced and significantly upregulated in tobacco leaves overexpressing the *AhRRS5* or *AhRLK1* gene [30, 31]. SA signaling is usually associated with *R* gene-mediated disease resistance and induces the expression of several *CML* genes [54–56]. Although the SA and JA defense pathways are usually antagonistic, synergistic functions have also been found in the defense response to pathogens [57–59]. These lines of evidence suggest that the *AhCML69* gene is involved in the HR, PTI, JA and SA pathways as a positive regulatory factor that modulates resistance to *R. solanacearum*.

## Conclusions

In the present study, 67, 72, and 214 *CaM/CML* genes were identified in *A. duranensis*, *A. ipaensis*, and *A. hypogaea*, respectively, and were divided into nine subgroups (Groups I-IX). There were 81 pairs of homologous genes in the *AhCaM/CML* gene family, and the Ka/Ks ratios of these gene pairs were all less than 1.0. The gene duplication events of the *AhCaM/CML* genes included whole-genome duplication, tandem repeats, and scattered repeats. Expression analysis revealed that *AhCML69* was constitutively expressed in the roots, stems, leaves, and flowers of peanut plants and was involved in the response to *R. solanacearum* infection. The AhCML69 protein was localized in the cytoplasm and nucleus. Transient overexpression of *AhCML69* in tobacco leaves increased resistance to *R. solanacearum* infection and induced the expression of defense-related genes, suggesting that *AhCML69* is a positive regulator of PTI by mediating the JA and SA pathways.

## Materials and methods

### Identification of the CaM/CML gene family

The genomes of the cultivated species *(A. hypogaea*, Shitouqi) and the wild species (*A. duranensis* and *A. ipaensis*) were retrieved and downloaded from the corresponding websites [34, 60]. Seven AtCaM and fifty *Arabidopsis* AtCML proteins from the TAIR (www.arabidopsis.org) database were subjected to BLASTP (E value < 1e-5) searches against the protein sequences from the *A. hypogaea*, *A. duranensis* and *A. ipaensis* genome databases. The EF-hand domain was characterized using SMART (http://smart.embl-heidelberg.de/) and InterPro (http://www.ebi.ac.uk/interpro/). A self-BLAST analysis of the CaM/CML proteins was performed to remove redundant protein sequences with coding sequences less than 1 kb in length, and the remaining sequences were considered putative CaM/CML proteins for further analysis. The molecular weights (MWs) and isoelectric points (pIs) of the CaM/CML proteins were predicted via the Ensembl Genome Browser and ProtComp 9.0 (http://linux1.softberry.com/).

### Chromosomal localization, gene structure and conserved motif analysis

The length information and location information of the *CaM/CML* genes were obtained from the GFF3 files on the genome database. The latest TBtools software [61] was employed to analyze the gene structures and chromosomal localization. The conserved motifs analysis of CaM/CML proteins were performed using the MEME program (http://memeesuite.org/tools/meme) with the default parameters.

### Multiple sequence alignments, phylogenetic analysis and collinearity analysis

Multiple sequence alignments were carried out using the ClustalW tool. A maximum likelihood (ML) tree was constructed by MEGA 11 software based on the full length of the CaM/CML protein sequences to investigate the evolutionary relationship among CaM/CML proteins. The collinearity relationships of the CaM/CML genes were analyzed by the Multiple Collinearity Scan toolkit MCScanX (http://chibba.pgml.uga.edu/mcscan2/) within the *A. hypogaea* genome and between homologous proteins in the *A. duranensis* and *A. ipaensis* genomes. The results were visualized using TBtools. The Ka/Ks ratios between *AhCaM/CML* members was calculated by KaKs_Calculator2.0 software [62].

### Plant materials and methods

The *N. benthamiana* seeds were evenly sown in a pot with nutrient soil. After the expansion of the third true leaf, one seedling was transferred into one pot and subsequently grown in a greenhouse at 28 ± 2 ℃ under a 16 h/8 h (light/dark) photoperiod. Four weeks later, these plants were subjected to transient expression assays. Peanut plants were cultivated according to our previous study [63]. Different tissues of peanut plants with seven to eight fully grown leaves were used for RNA extraction.

### Gene cloning and vector construction

The full CDSs of the *AhCML* genes were retrieved from our transcript data for peanut cultivars infected with *R.* *solanacearum* [33], and the primers for cloning were designed using SnapGene 4.1.8 (Additional File 10). The first-strand cDNA of peanut A165 [33], which was infected with *R.* *solanacearum*, was chosen as the amplification template for cloning the *AhCML* genes. The cloned genes were ligated to the PC2300-35S-eGFP vector and transformed into *Escherichia coli* DH5α. The PCR products of PC2300-35S-*AhCML69*/*AhCML174*-eGFP were recovered for gel detection and sent to Guangzhou Qingke Biotechnology Co., Ltd., for sequencing. The verified PC2300-*AhCML69*/*AhCML174*-eGFP vectors were subsequently transformed into *Agrobacterium tumefaciens* GV3101 for transient expression analysis.

### Transient expression and R. solanacearum infection

*A. tumefaciens* cells harboring the PC2300-35S-*AhCML69*/*AhCML174*-eGFP construct were grown overnight in LB liquid medium (10 g/L tryptone, 5 g/L yeast extract, 10 g/L NaCl, 1 mL/L kanamycin (50 mg/mL), 1 mL/L rifampicin (50 mg/mL)) at 28 °C. Bacterial cells were centrifuged and resuspended in infiltration buffer (10 mM MES, 10 mM MgSO_4_, and 200 mM acetosyringone, pH 5.6). The OD_600_ of the resuspended liquid was adjusted to 0.1. *A. tumefaciens* containing the empty vector or the PC2300-35S-AhCML69/AhCML174-eGFP vector was infiltrated into two sides of the same leaf using a 1.0 mL sterile syringe. Two different leaves of one *N.* *benthamiana* plant were used. The infiltrated plants were cultured in a greenhouse and subjected to further analysis at different timepoints. *R.* *solanacearum* cells were prepared according to the methods of a previous study [63]. *N. benthamiana* was inoculated with *R.* *solanacearum* at 48 h after infiltration with *A.* *tumefaciens*.

### Electrolyte leakage, trypan blue and DAB staining

Six leaves of three *N.* *benthamiana* plants were used for the electrolyte leakage measurement, trypan blue staining, and DAB histochemical analysis. Six pieces from every leaf were excised with a puncher of 6 mm diameter for electrolyte leakage measurement. The detailed procedures were described in our previous study [33]. For trypan blue, the six leaves were boiled for 2 min in trypan blue solution and incubated overnight in the dark at 28 °C. The stained leaves were destained in chloral hydrate solution (1.25 g/mL) at 25 °C and 50 r/min. The solution was changed every 3 h until the leaves were completely colorless, and photos of the leaves were taken. For DAB staining, the six leaves were subjected to vacuum for 2 min at 0.8 MPa in DAB staining solution and incubated overnight at 28 °C. These leaves were transferred into 90% ethanol to boil until completely colorless. These treated leaves can be preserved in absolute ethanol for a long period of time.

### Total RNA extraction and expression profiles

The leaves were collected from transiently transformed *N.* *benthamiana* plants or plants inoculated with *R.* *solanacearum* at 0, 24, 48, 72 and 96 h after infiltration. Different tissues of peanut plants were sampled from the cultivar Zhongkaihua 1. Total RNA was extracted using a HiPure Plant RNA Mini Kit B (Magen Biotechnology, Guangzhou, China) in accordance with the manufacturer’s instructions. The RNA from peanut tissues and *N.* *benthamiana* leaves was reverse transcribed to synthesize first-strand cDNA for RT‒qPCR according to the instructions of the EasyScript One-Step gDNA Removal and cDNA Synthesis SuperMix (TransGen Biotechnology, Beijing, China). RT‒qPCR analysis was performed according to the methods of a previous study [33]. The immune-related marker genes used in this study included the HR marker genes *NbH1N1* and *NbHsr203J*, the PTI marker genes *NbWIPK* and *NbPTI5*, the JA signaling pathway genes *NbOPR3* and *NbLOX*, and the SA signaling pathway genes *NbPR1* and *NbPR2*. *NbEF1a* and *Ahactin* were used as the internal reference genes for *N.* *benthamiana* and peanut, respectively. The primers used for RT‒qPCR are listed in Additional File 10.

The expression data for the representative *AhCML* genes in different tissues were downloaded from the Peanut Genome Resource (PGR) website (http://peanutgr.fafu.edu.cn/). Transcriptome data were obtained from our previous study [33]. Heatmaps of the expression profile values of the *AhCML* genes were generated with TBtools.

### Subcellular localization

Transient expression of *AhCML69* in *N.* *benthamiana* was performed according to the aforementioned protocol. Two days later, the infiltrated leaves were cut and observed under a Leica TCS SP8 confocal laser microscope (Leica Microsystems (Shanghai) Trading Co., Ltd., Mannheim, Germany). A wavelength of 488 nm was used for GFP excitation, and the 510 nm wavelength of the emission signal was obtained for the GFP channel. A wavelength of 640 nm was used for chlorophyll excitation, and 675 nm for the emission signal was used for the chlorophyll channel.

### Statistical analysis

Statistical analysis and graphs were performed by using the GraphPad Prism 8.0 software.

### Supplementary Information

**Supplementary Material 1.**

**Supplementary Material 2.**

**Supplementary Material 3.**

**Supplementary Material 4.**

**Supplementary Material 5.**

**Supplementary Material 6.**

**Supplementary Material 7.**

**Supplementary Material 8.**

**Supplementary Material 9.**

**Supplementary Material 10.**

## Data Availability

Expression data in different tissues can be downloaded from Peanut Genome Resource (PGR) website (http://peanutgr.fafu.edu.cn/). Transcriptomic data for the resistant and susceptible peanut leaves infected with *R. solanacearum* can be found in online repositories. The names of the repository/repositories and accession number(s) can be found below: NCBI – PRJNA861998.
