# Supplementary material for: Genome-wide analysis of the peanut CaM/CML gene family reveals that the AhCML69 gene is associated with resistance to Ralstonia solanacearum
Source: BMC Genomics. 2024 Feb 21;25:200. doi: 10.1186/s12864-024-10108-5 (PMC10880322; doi:10.1186/s12864-024-10108-5)
Supplement: Supplementary file 2 — Supplementary Material 2. [file 12864_2024_10108_MOESM2_ESM.docx]

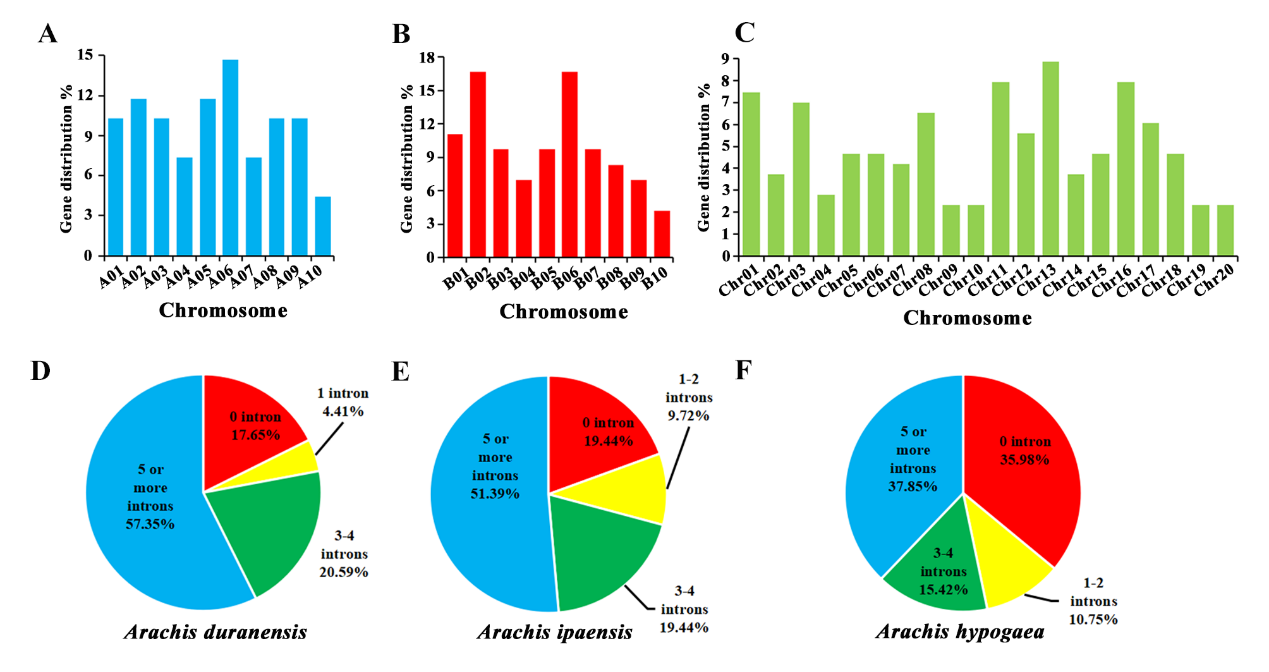


**Fig. S1** The intron numbers and distribution on chromosomes of *CaM/CML* genes in *A. hypogaea*, *A. duranensis* and *A. ipaensis*. **A-C** *CaM/CML* genes distribution on the chromosomes in *A. hypogaea*, *A. duranensis* and *A. ipaensis*, respectively. **D-F** Number of introns of *CaM/CML* genes in *A. hypogaea*, *A. duranensis* and *A. ipaensis*, respectively.
