# Supplementary material for: Genome-wide analysis of the peanut CaM/CML gene family reveals that the AhCML69 gene is associated with resistance to Ralstonia solanacearum
Source: BMC Genomics. 2024 Feb 21;25:200. doi: 10.1186/s12864-024-10108-5 (PMC10880322; doi:10.1186/s12864-024-10108-5)
Supplement: Supplementary file 9 — Supplementary Material 9. [file 12864_2024_10108_MOESM9_ESM.docx]

CDS:

>AH08G06260.1 + Chr08 11448317-11448805

ATGTTGTTTGCAAATATGACGTCATCAGTTCTAACTGCCACTGATTTGCGGCGGATATTT

GAGAAGCTTGACATGAATTGCGACGGTTTCGTGAGCCTTGAGGAACTCAATTGGCTCCTCCACAACATAGGCTTCCAATTAACGTTAGATGAATTGGAATCCCTTGTTGAGAAGAAAAGCCTTAACTTGAACGAGTTCTTGTTCTTCTATGACTCAATAATATCCAAGAAGAATTGCAATGATGAAGAGATGGATGATGAGTTGTTGGAGAATGATCTTGTGGAAACGTTCAAGGTGTTTGATTTGGATGGAGATGGATTCATAACGAGCAACGAGCTTCAGTGTGTTCTTAAGAGGCTTGGGTTGTGGGATGAGAGAGGTGGCAAGGATTGTGGTTCCATGATTCGATTCTATGATACCAATTCCGATGGCCAGCTTGATTTTGAGGAGTTTAAGAATATGATGTTGCTCACCATCACCACCGCATGA

>AH17G29650.1 - Chr17 124935811-124936719

ATGTTGTTTGCAAATATGACGTCATCAGTTCTAACTGCCACTGATTTGCGGCGGATATTT

GAGAAGCTTGACATGAATTGCGACGGTTTCGTGAGCCTTGAGGAACTCAATTGGCTCCTCCACAACATAGGCTTCCAATTAACGTTAGATGAATTGGAATCCCTTGTTGAGAAGAAAAGCCTTAACTTGAACGAGTTCTTGTTCTTCTATGACTCAATAATATCCAAGAAGAATTGCAATGACGACGATGATGATAATAATGAAGAGATGGATGATGAGTTGTTGGAGAATGATCTTGTGGAAACGTTCAAGGTGTTTGATTTGGATGGAGATGGATTCATAACGAGCAACGAGCTTCAGTGTGTTCTTAAGAGGCTTGGGTTGTGGGATGAGAGAGGTGGCAAGGATTGTGGTTCCATGATTCGATTCTATGATACCAATTCCGATGGCCAGCTTGATTTTGAGGAGTTTAAGAATATGATGTTCCTCACCATCACCACCGCATGA

Protein Sequence:

>AH08G06260.1

MLFANMTSSVLTATDLRRIFEKLDMNCDGFVSLEELNWLLHNIGFQLTLDELESLVEKKSLNLNEFLFFYDSIISKKNCNDEEMDDELLENDLVETFKVFDLDGDGFITSNELQCVLKRLGLWDERGGKDCGSMIRFYDTNSDGQLDFEEFKNMMLLTITTA*

>AH17G29650.1

MLFANMTSSVLTATDLRRIFEKLDMNCDGFVSLEELNWLLHNIGFQLTLDELESLVEKKSLNLNEFLFFYDSIISKKNCNDDDDDNNEEMDDELLENDLVETFKVFDLDGDGFITSNELQCVLKRLGLWDERGGKDCGSMIRFYDTNSDGQLDFEEFKNMMFLTITTA*
